# 4-Chloro­anilinium bromide

**DOI:** 10.1107/S1600536812024993

**Published:** 2012-06-13

**Authors:** Min-Min Zhao

**Affiliations:** a College of Chemistry and Chemical Engineering, Southeast University, Nanjing 210096, P. R. China

## Abstract

In the title compound, C_6_H_7_ClN^+^·Br^−^, the amino N atom is protonated. All non-H atoms of the cation are essentially coplanar [r.m.s. deviation = 0.004 (3) Å]. In the crystal, N—H⋯Br hydrogen bonds connect the ions, forming a ribbon-like structure propagating along [010].

## Related literature

For the structures and properties of related compounds, see: Fu *et al.* (2011*a* ▶,*b* ▶,*c* ▶); Wang *et al.* (2002 ▶); Xue *et al.* (2002 ▶); Ye *et al.* (2008 ▶).
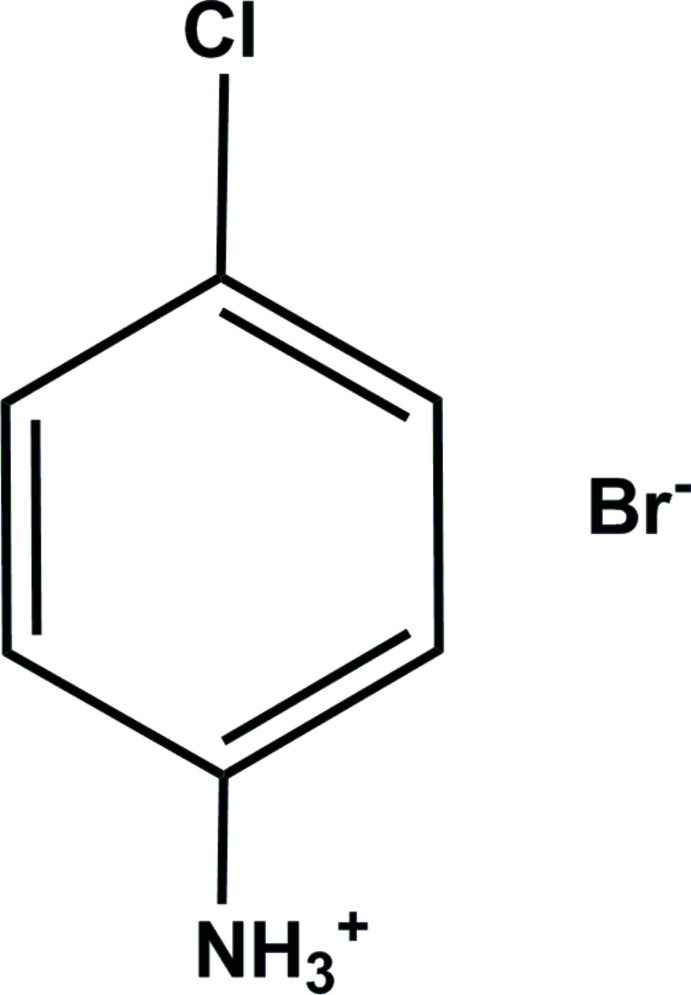


## Experimental

### 

#### Crystal data

C_6_H_7_ClN^+^·Br^−^
*M* *_r_* = 208.49
Triclinic, 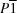

*a* = 4.3989 (2) Å
*b* = 6.2553 (2) Å
*c* = 13.8907 (8) Å
α = 91.4000 (8)°
β = 93.580 (1)°
γ = 101.967 (1)°
*V* = 372.91 (3) Å^3^
*Z* = 2
Mo *K*α radiation
μ = 5.78 mm^−1^
*T* = 153 K
0.10 × 0.05 × 0.05 mm

#### Data collection

Rigaku Mercury CCD diffractometer
Absorption correction: multi-scan (*CrystalClear*; Rigaku, 2005 ▶) *T*_min_ = 0.910, *T*_max_ = 1.000
3843 measured reflections
1647 independent reflections
1435 reflections with *I* > 2σ(*I*)
*R*_int_ = 0.056

#### Refinement

*R*[*F* ^2^ > 2σ(*F* ^2^)] = 0.041
*wR*(*F* ^2^) = 0.103
*S* = 1.11
1647 reflections
83 parameters
H-atom parameters constrained
Δρ_max_ = 0.84 e Å^−3^
Δρ_min_ = −1.47 e Å^−3^

### 

Data collection: *CrystalClear* (Rigaku, 2005 ▶); cell refinement: *CrystalClear*; data reduction: *CrystalClear*; program(s) used to solve structure: *SHELXS97* (Sheldrick, 2008 ▶); program(s) used to refine structure: *SHELXL97* (Sheldrick, 2008 ▶); molecular graphics: *SHELXTL* (Sheldrick, 2008 ▶); software used to prepare material for publication: *SHELXTL*.

## Supplementary Material

Crystal structure: contains datablock(s) I, global. DOI: 10.1107/S1600536812024993/aa2066sup1.cif

Structure factors: contains datablock(s) I. DOI: 10.1107/S1600536812024993/aa2066Isup2.hkl

Supplementary material file. DOI: 10.1107/S1600536812024993/aa2066Isup3.cml

Additional supplementary materials:  crystallographic information; 3D view; checkCIF report

## Figures and Tables

**Table 1:** Hydrogen-bond geometry (Å, °)

*D*—H⋯*A*	*D*—H	H⋯*A*	*D*⋯*A*	*D*—H⋯*A*
N1—H1*A*⋯Br1^i^	0.89	2.59	3.467 (4)	167
N1—H1*B*⋯Br1^ii^	0.89	2.52	3.370 (4)	161
N1—H1*C*⋯Br1^iii^	0.89	2.46	3.312 (4)	161

Symmetry codes: (i) 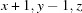; (ii) 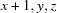; (iii) 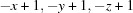.

**Fig. 1.**
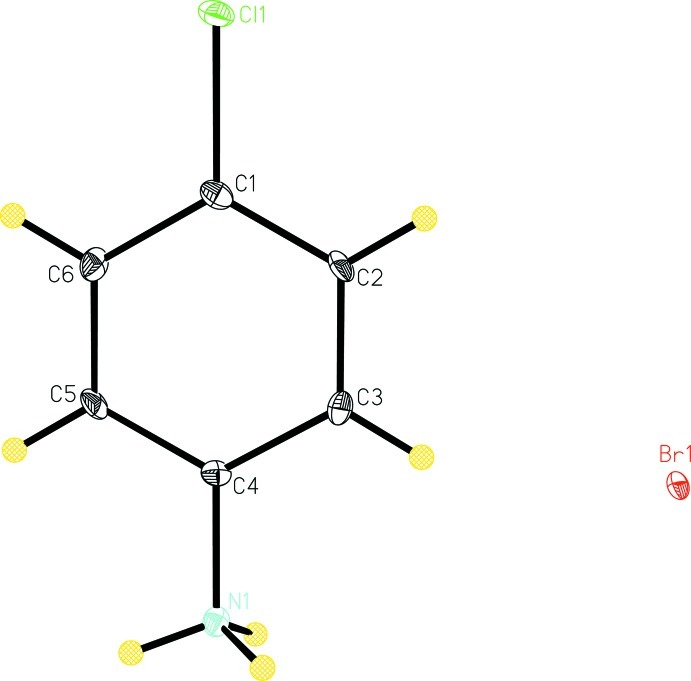
A view of the asymmetric unit with the atomic numbering scheme. The displacement ellipsoids were drawn at the 30% probability level.

**Fig. 2.**
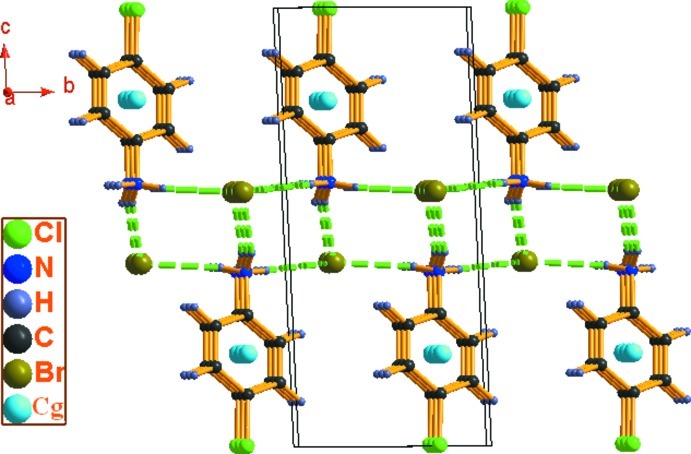
The crystal packing of the title compound viewed along the a axis showing the H–bonding and π–π interactions (dashed line), Cg is the centroid of the benzene ring.

## supplementary crystallographic information

### Comment

Simple organic salts containing strong intrermolecular H–bonds have attracted an attention as materials which display ferroelectric–paraelectric phase transitions (Fu *et al.*, 2011*a*, *b*, *c*). With the purpose of obtaining phase transition crystals of organic salts, various organic molecules have been studied and a series of new crystal materials have been elaborated (Wang *et al.*, 2002; Xue *et al.*, 2002; Ye *et al.*, 2008).

In the title compound (Fig. 1), the bond lengths and angles have normal values. The asymmetric unit is composed of one 4-chloroanilinium cation and one Br^-^ anion. The protonated N atom is involved in strong intermolecular N—H···Br hydrogen bonds (Table 1) which connect the ions into a 2D network parallel to the *ab*–plane (Fig. 2). The crystal packing is further stabilized by aromatic π–π interactions between the benzene rings of the neighbouring cations with the Cg···Cg distances of 4.399 (1)Å (Cg is the centroid of the benzene ring).

### Experimental

The HBr (1 mL, 2 mol/L), 4-chloroaniline (10 mmol) and ethanol (50 mL) were added into a 100 mL flask. The mixture was stirred at 333 K for 2 h, and then the precipitate was filtered out. Colourless crystals suitable for X–ray diffraction were obtained by slow evaporation of the solution.

### Refinement

All the H atoms attached to C atoms were placed into the idealized positions and treated as riding with C—H = 0.93Å (aromatic) and *U_iso_*(H) = 1.2*U_eq_*(C). The H atoms based on N were placed into the calculated positions with the N—H = 0.89Å and refined with *U_iso_*(H) = 1.5*U_eq_*(N).

### Crystal data

**Table:**

C_6_H_7_ClN^+^·Br^−^	*Z* = 2
*M**_r_* = 208.49	*F*(000) = 204
Triclinic, *P*1	*D*_x_ = 1.857 Mg m^−^^3^
Hall symbol: -P 1	Mo *K*α radiation, λ = 0.71073 Å
*a* = 4.3989 (2) Å	Cell parameters from 1674 reflections
*b* = 6.2553 (2) Å	θ = 3.0–27.5°
*c* = 13.8907 (8) Å	µ = 5.78 mm^−^^1^
α = 91.4000 (8)°	*T* = 153 K
β = 93.580 (1)°	Block, colourless
γ = 101.967 (1)°	0.10 × 0.05 × 0.05 mm
*V* = 372.91 (3) Å^3^	

### Data collection

**Table:**

Rigaku Mercury CCD diffractometer	1647 independent reflections
Radiation source: fine-focus sealed tube	1435 reflections with *I* > 2σ(*I*)
Graphite monochromator	*R*_int_ = 0.056
Detector resolution: 13.6612 pixels mm^-1^	θ_max_ = 27.5°, θ_min_ = 2.9°
CCD profile fitting scans	*h* = −5→5
Absorption correction: multi-scan (*CrystalClear*; Rigaku, 2005)	*k* = −8→8
*T*_min_ = 0.910, *T*_max_ = 1.000	*l* = −18→18
3843 measured reflections	

### Refinement

**Table:**

Refinement on *F*^2^	Primary atom site location: structure-invariant direct methods
Least-squares matrix: full	Secondary atom site location: difference Fourier map
*R*[*F*^2^ > 2σ(*F*^2^)] = 0.041	Hydrogen site location: inferred from neighbouring sites
*wR*(*F*^2^) = 0.103	H-atom parameters constrained
*S* = 1.11	*w* = 1/[σ^2^(*F*_o_^2^) + (0.044*P*)^2^] where *P* = (*F*_o_^2^ + 2*F*_c_^2^)/3
1647 reflections	(Δ/σ)_max_ < 0.001
83 parameters	Δρ_max_ = 0.84 e Å^−^^3^
0 restraints	Δρ_min_ = −1.47 e Å^−^^3^

### Special details

**Table:**

Geometry. All s.u.'s (except the s.u. in the dihedral angle between two l.s. planes) are estimated using the full covariance matrix. The cell s.u.'s are taken into account individually in the estimation of s.u.'s in distances, angles and torsion angles; correlations between s.u.'s in cell parameters are only used when they are defined by crystal symmetry. An approximate (isotropic) treatment of cell s.u.'s is used for estimating esds involving l.s. planes.
Refinement. Refinement of F^2^ against ALL reflections. The weighted R-factor wR and goodness of fit S are based on F^2^, conventional R-factors R are based on F, with F set to zero for negative F^2^. The threshold expression of F^2^ > 2sigma(F^2^) is used only for calculating R-factors(gt) etc. and is not relevant to the choice of reflections for refinement. R-factors based on F^2^ are statistically about twice as large as those based on F, and R- factors based on ALL data will be even larger.

### Fractional atomic coordinates and isotropic or equivalent isotropic displacement parameters (Å^2^)

**Table:**

	*x*	*y*	*z*	*U*_iso_*/*U*_eq_	
Cl1	0.6759 (3)	0.27570 (19)	0.99710 (9)	0.0205 (3)	
N1	1.1049 (9)	0.2261 (6)	0.6017 (3)	0.0136 (8)	
H1A	1.2142	0.1217	0.5980	0.020*	
H1B	1.2258	0.3545	0.5904	0.020*	
H1C	0.9437	0.1973	0.5580	0.020*	
C1	0.7915 (11)	0.2566 (8)	0.8806 (4)	0.0154 (10)	
Br1	0.41356 (10)	0.76145 (7)	0.58041 (3)	0.01335 (18)	
C2	0.7543 (11)	0.4182 (7)	0.8164 (3)	0.0155 (10)	
H2A	0.6637	0.5329	0.8352	0.019*	
C3	0.8537 (11)	0.4061 (7)	0.7241 (4)	0.0151 (10)	
H3A	0.8289	0.5113	0.6798	0.018*	
C4	0.9907 (10)	0.2340 (7)	0.6991 (3)	0.0113 (9)	
C5	1.0258 (11)	0.0722 (7)	0.7620 (3)	0.0151 (10)	
H5A	1.1156	−0.0428	0.7430	0.018*	
C6	0.9254 (11)	0.0842 (7)	0.8533 (4)	0.0159 (10)	
H6A	0.9471	−0.0233	0.8968	0.019*	

### Atomic displacement parameters (Å^2^)

**Table:**

	*U*^11^	*U*^22^	*U*^33^	*U*^12^	*U*^13^	*U*^23^
Cl1	0.0316 (7)	0.0169 (6)	0.0146 (6)	0.0075 (5)	0.0080 (5)	−0.0018 (5)
N1	0.018 (2)	0.011 (2)	0.012 (2)	0.0037 (16)	−0.0009 (17)	−0.0014 (16)
C1	0.017 (2)	0.014 (2)	0.015 (3)	0.0028 (19)	0.002 (2)	−0.0045 (19)
Br1	0.0165 (3)	0.0087 (3)	0.0154 (3)	0.00395 (18)	0.00213 (18)	−0.00161 (17)
C2	0.020 (2)	0.011 (2)	0.018 (3)	0.0072 (19)	0.004 (2)	−0.0033 (19)
C3	0.018 (2)	0.009 (2)	0.019 (3)	0.0018 (19)	0.001 (2)	0.0029 (19)
C4	0.013 (2)	0.009 (2)	0.010 (2)	−0.0007 (17)	0.0002 (18)	−0.0041 (18)
C5	0.019 (2)	0.011 (2)	0.017 (3)	0.0065 (19)	0.003 (2)	−0.0043 (19)
C6	0.023 (3)	0.008 (2)	0.016 (3)	0.0023 (19)	−0.001 (2)	0.0000 (19)

### Geometric parameters (Å, º)

**Table:**

Cl1—C1	1.735 (5)	C2—H2A	0.9300
N1—C4	1.476 (6)	C3—C4	1.385 (6)
N1—H1A	0.8900	C3—H3A	0.9300
N1—H1B	0.8900	C4—C5	1.379 (6)
N1—H1C	0.8900	C5—C6	1.375 (7)
C1—C6	1.388 (6)	C5—H5A	0.9300
C1—C2	1.392 (6)	C6—H6A	0.9300
C2—C3	1.385 (7)		
			
C4—N1—H1A	109.5	C4—C3—C2	118.6 (4)
C4—N1—H1B	109.5	C4—C3—H3A	120.7
H1A—N1—H1B	109.5	C2—C3—H3A	120.7
C4—N1—H1C	109.5	C5—C4—C3	122.5 (4)
H1A—N1—H1C	109.5	C5—C4—N1	119.2 (4)
H1B—N1—H1C	109.5	C3—C4—N1	118.3 (4)
C6—C1—C2	120.9 (5)	C6—C5—C4	118.7 (4)
C6—C1—Cl1	119.8 (4)	C6—C5—H5A	120.6
C2—C1—Cl1	119.2 (4)	C4—C5—H5A	120.6
C3—C2—C1	119.3 (4)	C5—C6—C1	120.0 (4)
C3—C2—H2A	120.4	C5—C6—H6A	120.0
C1—C2—H2A	120.4	C1—C6—H6A	120.0

### Hydrogen-bond geometry (Å, º)

**Table:**

*D*—H···*A*	*D*—H	H···*A*	*D*···*A*	*D*—H···*A*
N1—H1*A*···Br1^i^	0.89	2.59	3.467 (4)	167
N1—H1*B*···Br1^ii^	0.89	2.52	3.370 (4)	161
N1—H1*C*···Br1^iii^	0.89	2.46	3.312 (4)	161

Symmetry codes: (i) *x*+1, *y*−1, *z*; (ii) *x*+1, *y*, *z*; (iii) −*x*+1, −*y*+1, −*z*+1.
